# Effects of Gametophytes of *Ecklonia Kurome* on the Levels of Glucose and Triacylglycerol in *db/db*, Prediabetic C57BL/6J and IFN-γ KO Mice

**Published:** 2012-03

**Authors:** Febriza Dwiranti, Masanori Hiraoka, Takahiro Taguchi, Yuko Konishi, Mari Tominaga, Akira Tominaga

**Affiliations:** 1 *Division of Human Health and Medical Science, Graduate School of Kuroshio Science, Kochi University, Kohasu, Okoh-cho, Nankoku, Kochi 783-8505 Japan;*; 2 *Usa Marine Biological Institute, Kochi University, 194 Inoshiri, Usa, Tosa, Kochi 781-1164 Japan;*; 3 *Life Science and Functional Materials Section, Science Research Center, Kochi University, Kohasu, Okoh-cho, Nankoku, Kochi 783-8505, Japan;*; 4 *Department of Medical Technology, Kochi Gakuen College, 292-26 Asahitenjin-cho, Kochi, Kochi 780-0955, Japan*

**Keywords:** *db/db* mice, diabetes, *Ecklonia kurome*, gametophytes, glucose, IFN-γ, IFN-γ KO mice, leptin

## Abstract

We have studied edible algae that have the potential to down-regulate blood glucose. In Japan, *Ecklonia* species have been believed to improve the circulation of blood. In this study, we used leptin receptor deficient type 2 diabetes model mice (*db/db*) and prediabetic C57BL/6J mice. We also focused on the role of IFN-γ in the control of blood levels of triacylglycerol and glucose, because it is reportedly engaged in the regulation of energy consumption together with leptin. We report that gametophytes of *Ecklonia kurome* down-regulate the blood level of glucose and serum level of triacylglycerol in *db/db*. We also report that gametophytes of *Ecklonia kurome* down-regulate the level of glucose but not the level of triacylglycerol in prediabetic C57BL/6J mice induced by a high fat diet. They increased the level of triacylglycerol compared to that of control group in C57BL/6J, but not in IFN-γ KO mice. Gametophytes of *Ecklonia kurome* were administered orally to prediabetic C57BL/6J and IFN-γ KO mice and oral glucose tolerance tests were performed to evaluate the effects of algae. During the administration of the normal diet, we found a higher level of blood glucose in a glucose tolerance test of IFN-γ KO mice compared with that of C57BL/6J. Although a high fat diet induced a higher level of blood glucose compared with a normal diet group in a glucose tolerance test of C57BL/6J mice, this effect of high fat diet was not observed clearly at first but appeared three hours after glucose administration in IFN-γ KO mice. Gametophytes of *Ecklonia kurome* down-regulated the level of blood glucose in both C57BL/6J and IFN-γ KO mice, when administered a normal diet after making them prediabetic. These results suggest that *Ecklonia kurome* are effective to down-regulate the blood glucose and IFN-γ is involved in the regulation of blood glucose and triacylglycerol.

## INTRODUCTION

The world prevalence of diabetes has been reported on and it is estimated that more than 400 million adults will suffer from diabetes by 2030 (1). It has also been reported that the prevalence of type 2 diabetes has reached epidemic levels in Asia and that this epidemic threatens to overwhelm health-care systems in the region (2). Due to the lifestyle changes caused by mechanization and urbanization, high nutrient availability and an energy dense diet along with decreasing physical activity levels have predisposed people to both obesity and type 2 diabetes (2, 3). It has been suggested that certain changes in lifestyle can be effective in the prevention of type 2 diabetes, especially for those with impaired glucose tolerance (2).

In Asian countries, algae have traditionally been considered as a food good for health. In Japan, algae are believed to reduce blood pressure or to ameliorate atherosclerosis. Indeed, bio-active compounds from edible algae have been reported not only in the prevention of hypertention but also in anti-diabetic activities. It is reported that fucoxanthin (4) and phlorotannins (5) have anti-diabetic activities.

Here, we tried to find a good edible alga which can be used as a diet. So, we focused on the anti-diabetic effect of algae found in the area where we live. A brown alga, *Ecklonia kurome* (*E. kurome*), a green alga, *Ulva* species, a red alga *Porphyra* species, and a filamentous blue-green alga were collected off Shikoku Island, west part of Japan. Especially, *E. kurome* has been believed to improve the circulation of blood. Furthermore, we focused on the role of gametophytes of *E. kurome*, because the conversion of fatty acids to carbohydrates is manipulated to regulate the energy consumption which may be important before fertilization. We also used a brown alga, *Cladosiphon okamuranus*, Okinawa Mozuku grown in Okinawa island and a blue-green alga, *Spirulina pacifica* grown in Hawaii island. It is reported that *Spirulina* supplementation lowered plasma triacylglycerol significantly in type 2 diabetes mellitus patients (6).

The precise function of these algae, however, has not been well examined. Here, we have focused on the regulatory effect of algae on the development of type 2 diabetes. At first, we examined the effects of various algae on the blood glucose level in leptin receptor deficient *db/db* mice (7-9) and found that gametophytes of *E. kurome* down-regulate blood glucose and serum triacylglycerol. Sporophytes of *E. kurome* did not have any effects on these levels. Next, to examine the effect of gametophytes of *E. kurome* in wild type mice, we established the prediabetic status of C57BL/6J by administrating a high fat diet. Although dietary interventions in humans showed no consistent deleterious effects of high fat diets on insulin sensitivity (10, 11), it has been reported that with the exception of omega-3 fatty acids diets high in saturated, monounsaturated (omega-9), or polyunsaturated (omega-6) fatty acids have led to severe insulin resistance in rats (12). We established prediabetic status in C57BL/6J by administrating a high fat diet containing mainly monounsaturated and saturated fatty acids. We found that gametophytes of *E. kurome* down-regulate blood glucose in prediabetic C57BL/6J wild type mice whose condition was induced by administrating a high fat diet (HFD). However, the serum level of triacylglycerol was not down-regulated as in *db/db* mice.

It has been reported that leptin increases Th1 cells which secrete IFN-γ and suppress Th2 cytokine production (13). Leptin is also reported to down-regulate the regulatory T cells (Treg) and stimulate the proliferation of IFN-γ secreting Th1 cells (14). We established prediabetic status in IFN-γ KO mice that have a C57BL/6J genetic background (15) to examine the effect of gametophytes of *E. kurome* on the level of blood glucose.

IFN-γ produced by Th1 cells is a cytokine involved in inflammatory responses that induces fever and enhances thermogenesis. Since type 2 diabetes is caused by high calorie intake and less energy consumption, we hypothesized that both leptin and IFN-γ are engaged in the occurrence of this disease. The reason we used leptin receptor deficient *db/db* mice and IFN-γ KO mice is that we may get clear results by examining the effect of one cytokine in the absence of the other cytokine, when two cytokines are involved in the regulation of lymphocytes and glucose metabolism.

In this study, we will discuss the potential usefulness of gametophytes of *E. kurome* as an alternative medicine in down-regulating blood glucose by regulating the immune system including cytokines such as IFN-γ or leptin.

## MATERIALS AND METHODS

### Animals

Female leptin receptor deficient, BKS.Cg-+*Lepr^db^/*+*Leptr^db^*/Jcl (*db/db*) mice (7-9) were obtained from CLEA Japan (Tokyo, Japan) at six weeks of age. Female C57BL/6J mice were obtained at six weeks of age from CLEA Japan and maintained for two weeks before the start of experiments in our animal facility. IFN-γ KO, C57BL/6J-*Ifng^tm1Ts^* mice (15) were obtained from Dr. Leonard Shultz at the Jackson Laboratory and maintained in HEPA filter-passed air in the Animal Facility of Kochi University. All experiments were performed under SPF conditions. This study is approved by the Animal Care and Use Committee for Kochi University. The schedule of experiments are described in Table 1. Body weights were recorded and summarized in Table 2.

### Preparation and administration of algae

A brown alga *Ecklonia kurome* (*E. kurome*), a green alga *Ulva* species which was previously reported as E16 strain of *Ulva* sp.2 by Shimada *et al.* (16), a red alga *Porphyra* species (*Porphyra* sp.), and a filamentous blue-green alga (fil. alga, species was not identified) were collected off Shikoku Island, Japan and isolated as unialgal strains respectively. The isolated strains have been kept as culture collections in Usa Marine Biological Institute, Kochi University. The thalli of *E. kurome, Ulva* species and *Porphyra* species were cultured in outdoor tanks using the “germling cluster” method and deep seawater supply developed by Hiraoka and Oka (17). The culture method led to a stable mass production of the seaweeds all the year around. The deep seawater pumped up from a depth of over 300 m in the east coast of Muroto Cape, Shikoku Island has properties appropriate for the stable seaweed cultivation, of which temperature and quantities of major inorganic nutrients (nitrate and phosphate) are constant throughout the year (Hiraoka and Oka, 17). The blue-green alga and the micro gametophytes of *E. kurome* were cultured in a glass flask containing 500 mL ES medium (Autoclaved natural seawater containing 8.26 × 10^-4^ M Tris base, 8.24 × 10^-4^ M NaNO_3_, 4.63 × 10^-5^ M Na_2_ β-glycerophosphate, 2.84 × 10^-5^ M Na_2_EDTA, 8.95 × 10^-6^ M Fe(NH_4_)_2_(SO_4_)_2_, 8.95 × 10^-7^ M FeCl_3_, 3.64 × 10^-6^ M MnSO_4_, 3.82 × 10^-7^ M ZnSO_4_, 8.48 × 10^-8^ M CoSO_4_, 2.96 × 10^-8^ M thiamine, 4.09 × 10^-10^ M biotin, 1.48 × 10^-10^ M cyanocobalamin: Provasoli, 18) at 25°C with a 12:12 L:D cycle under cool white fluorescent light (approximately 100 µmol photons m^-2^s^-1^). The medium was exchanged weekly. Thalli of a brown alga *Cladosiphon okamuranus* (*C. okamuranus*), called Okinawa Mozuku in Japanese, were cultivated by setting out culture nets in the coastal areas of Okinawa, Japan and donated to us by Mr. Ippei Yanagida. *Spirulina pacifica* (*S. pacifica*), a strain of edible *Spirulina* (*Arthrospira*) *platensis* was a gift from Dr. Gerald Cysewski, Cyanotech Cooperation (Kailua-Kona, Hawaii) and Mr. Nobuyuki Miyaji, Toyo Koso Kagaku Co., LTD, (Urayasu, Chiba, Japan).

All algae were suspended in distilled water (DW) at a concentration of 50 mg/ml (wet weight). They were grinded in a mortar and homogenized using a Teflon homogenizer followed by homogenization with a Polytron (Kinematica, Luzern, Switzerland). Homogenate of algae was heated at 80°C for one hour and the procedure was repeated. All algae samples were administered orally using a polyethylene capillary. Ten milligrams of algae homogenate (in a volume of 0.2 ml of 50 mg/ml solution) were administered every other day to each mouse. As a control, DW (Otsuka Pharmaceutical Co., Ltd., Tokushima, Japan) was administered.

### Diets and reagents

Normal diet CE-2 and High Fat Diet 32 (HFD 32) were purchased from CLEA Japan. CE-2 contains 4.8% crude fat and HFD 32 contains 7.1% saturated fatty acids (4% palmitic acid and 2.4% stearic acid), 21.2% monounsaturated fatty acids (20.5% oleic acid), 3.3% polyunsaturated fatty acid (3.2% linoleic acid). HFD 32 contains 27.4% carbohydrates. *Db/db* mice were fed with CE-2. Leptin enzyme-linked immunosorbent assay (ELISA) Kit (AKRLP-011) and Insulin ELISA kit (AKRIN-011T) were purchased from Shibayagi Co., Ltd. (Shibukawa, Gunma, Japan).

### Oral glucose tolerance test (OGTT)

At end of each stage of the experimental schedule (Table 1), mice were fasted for 18 hours. Then, one drop of blood was collected from a tail vein of each mouse hourly (0, 1, 2, and 3 hours) after oral administration of glucose (2 g/kg body weight). Blood glucose was measured with Accu-Chek (Roche Diagnostics K.K., Tokyo, Japan) in all experiments.

**Table 1:** Schedule of administration of diets and algae

Group No. Strain	Stage 1 (8 weeks)	Stage 2 (4 weeks)	Stage 3 (3 weeks)	Stage 4 (3 weeks)

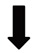 Serum				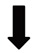
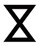 Flow cyto.				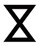
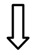 OGTT	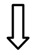	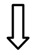	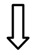	

1 (C57BL/6J)	ND (CE-2)	ND	ND	ND
2 (C57BL/6J)	HFD (HFD32)	HFD + DW	ND + DW	HFD + DW
3 (C57BL/6J)	HFD (HFD32)	HFD + gametophytes *E. kurome*	ND + gametophytes *E. kurome*	HFD + gametophytes *E. kurome*
4 (C57BL/6J)	HFD (HFD32)	HFD + sporophytes *E. kurome*	ND + sporophytes *E. kurome*	HFD + sporophytes *E. kurome*
5 (C57BL/6J)	HFD (HFD32)	HFD + *C. okamuranus*	ND + *C. okamuranus*	HFD + *C. okamuranus*
6 (C57BL/6J)	HFD (HFD32)	HFD + *Porphyra* sp.	ND + *Porphyra* sp.	HFD + *Porphyra* sp.
7 (IFN-γ KO)	ND (CE-2)	ND (CE-2)	ND (CE-2)	ND (CE-2)
8 (IFN-γ KO)	HFD (HFD32)	HFD + DW	ND + DW	HFD + DW
9 (IFN-γ KO)	HFD (HFD32)	HFD + gametophytes *E. kurome*	ND + gametophytes *E. kurome*	HFD + gametophytes *E. kurome*

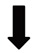
: Bleeding for serum collection; 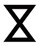: Bleeding for Flow cyto. (flow cytometry); 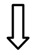: OGTT. OGTT was performed at the end of each stage as indicated. To prepare sera, 100 μL blood was collected from orbital sinus of each mouse eleven days before being sacrificed. Lymphocytes for flow cytometry were collected one day before being sacrificed and stained as described in Materials and methods. *E. kurome*: *Ecklonia kurome*, *C. okamuranus*: *Cladosiphon okamuranus*, *Porphyra* sp: species belonging to genus *Porphyra*. In groups 1, 7, 8, and 9, each group consists of 5 female mice. In groups from 2 to 6, each group consists of 8 female mice. From group 1 to 6, eight weeks old female C57BL/6J mice were used. IFN-γ KO mice were 8 to 15 weeks old female mice. All algae homogenate suspended in distilled water (DW) was administered using capillaries as described in Materials and methods.

### Criteria for diabetes and intermediate hyperglycemia

We defined the prediabetic status according to recommended diagnostic criteria by World Health Organization and International Diabetes Federation (19). Impaired Glucose Tolerance (intermediate hyperglycemia: prediabetic) was defined as follows: Fasting plasma glucose: <7.0 mmol/L and plasma glucose two hours after ingestion of 75 g glucose load (2-h plasma glucose): ≥7.8 and <11.1 mmol/L. Diabetes was defined as follows: Fasting plasma glucose: ≥7.0 mmol/L or 2-h plasma glucose ≥11.1 mmol/L.

### Measurement of serum levels of glucose, triacylglycerol, and cholesterol

**Figure 1:**
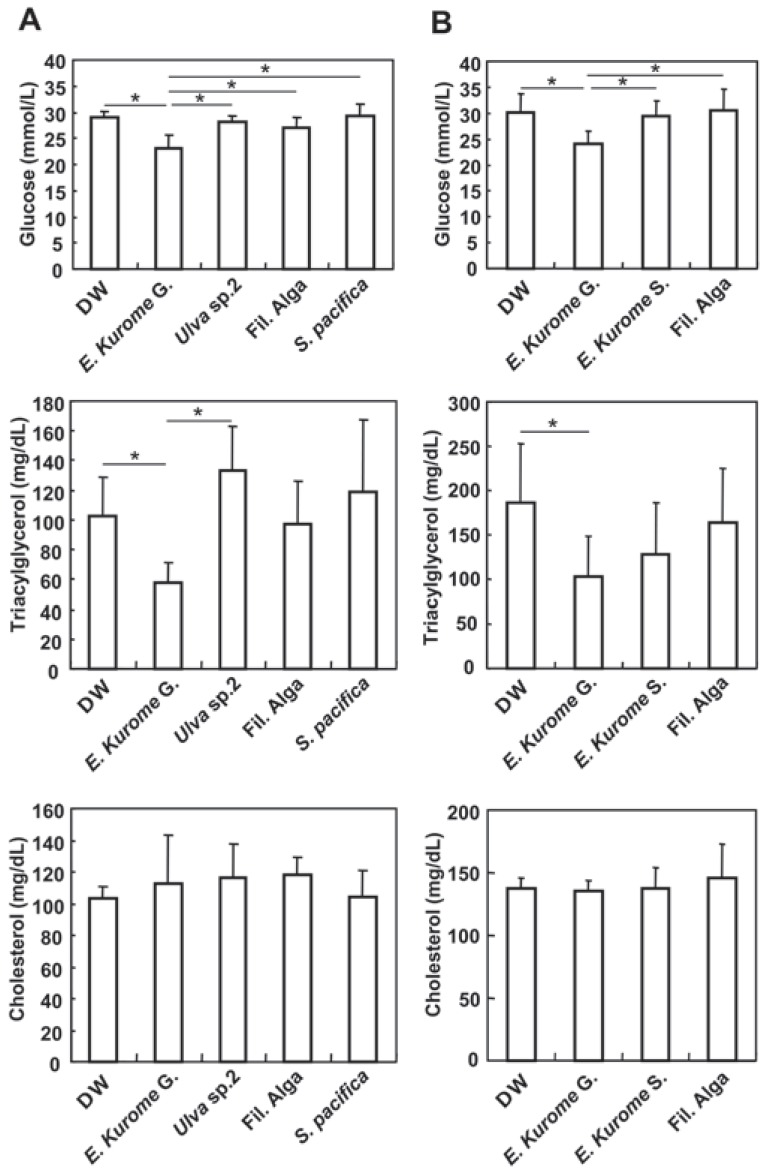
Effects of algae on blood level of glucose and serum levels of triacylglycerol and cholesterol in *db/db* mice. A. Gametophytes of *E. kurome,* green alga *Ulva* sp.2, and Fil. Alga (filamentous blue-green alga), and *S. pacifica* were administered to *db/db* mice orally starting at the age of 15 weeks. Algae homogenates were administered for 10 weeks. At the end of the administration of algae homogenates, sera were prepared as described in Materials and methods. Each group consisted of 5 female mice. Results are expressed as average ± S.D. (n=4); B. Gametophytes of *E. kurome* (*E. krurome* G), sporophytes of *E. kurome*, and Filamentous blue-green alga (Fil. Alga) were administered for 4 weeks. Results were expressed as average ± S.D. (n=8). Asterisks indicate that there are significant differences between groups indicated by a line. One way ANOVA, *P*<0.05.

Sera were prepared from the blood collected from orbital sinus using a glass capillary (Legend of Fig. 1 and Table 1). One hundred microliter of blood was collected from each mouse after being anesthetized. Serum levels of glucose, triacylglycerol, and cholesterol were measured by Hitachi Clinical Analyzer S40 (Hitachi, Ltd., Tokyo, Japan) using S-Test Cartridges for glucose, triacylglycerol, and cholesterol (Alfa Wassermann, Inc., West Caldwell, NJ).

### Measurements of insulin and leptin

Measurements of serum levels of insulin and leptin were performed according to the manufacturer’s manual. TMB (3, 3′, 5, 5′-tetramethylbenzidine) was used as a chromogen. The levels of both insulin and leptin in the serum were evaluated by measuring the absorbance at 450 nm using a microplate reader ThermoMax ROM v102 (Molecular Devices, Sunnyvale, CA).

### Flow cytometry

The percentage of Treg cells among lymphocytes of each group was evaluated from the pooled peripheral blood. Ten million lymphocytes were collected by using Lympholyte-M (Cedarlane, Hornby, Ontario, Canada). Treg cells were stained using Mouse peridinin chlorophyll protein (PerCP) conjugated anti-CD4 antibodies (PerCP-anti-CD4 Ab) and fluorescein isothiocyanate (FITC) or phycoerythrin (PE) conjugated anti-CD25 antibodies (FITC-anti-CD25 Ab or PE-anti-CD25 Ab) according to the manufacturer’s protocol (Bio Legend, San Diego, CA). Five thousands stained cells were analyzed with a FACS-Calibur (Becton Dickinson and Co., Mountain View, CA), using Quadra Stats of BD Cell Quest^TM^ Pro Version 5.2.

### Statistics

For the oral glucose tolerance test, an ANOVA was performed and significance was determined using Tukey-Kramer’s post hoc test. For serum glucose, triacylglycerol, cholesterol, leptin, insulin, adipose tissues, and body weights, one way ANOVA was used to evaluate the statistical significance. A *P* value <0.05 was considered statistically significant.

## RESULTS

In East Asian countries, several algae have traditionally been considered to improve the circulation of blood. In particular, in Japan, *Ecklonia* species have been believed to improve the properties of blood vessels by lowering blood pressure or by ameliorating atherosclerosis. As one of the functions, we examined the effects of algae on the control of blood glucose. At first, we administered algae homogenate to leptin receptor deficient *db/db* mice, well known models of type 2 diabetes (7-9). Since they are defective in leptin signal reception, they eat more than twice as much as food compared with normal mice, resulting in obesity and diabetes. When starting the administration of algae (15 weeks old, female), the average body weight of *db/db* mice was 56.6 g and the average blood glucose concentration was 25.3 mmol/L. The average uptake of food by *db/db* mice was 6.2 g pellets food/mouse/day, which is more than twice as much eaten by wild mice. We continuously served a normal diet (ND: CE-2, Materials and methods). High calorie foods and less exercises induced symptoms similar to diabetes mellitus type 2. Thus, after confirming the symptoms of diabetes, we administered the homogenized algae for 10 weeks and found that gametophytes of *E. kurome* down-regulated blood glucose and serum triacylglycerol significantly compared with the DW group or other algae groups (Fig. 1A). There was no significant difference among groups at the serum level of cholesterol.

Next, we examined whether only gametophytes in the life cycle of *E. kurome* had these effects in *db/db* mice. We administered either gametophytes or sporophytes of *E. kurome* and a filamentous blue-green alga to female *db/db* mice starting at the age of 15 weeks (average blood glucose: 27.7 mmol/L; average body weight: 58.6 g). Four weeks after administration of the algae, we measured the levels of blood glucose, serum triacylglycerol, and cholesterol. The level of blood glucose in the gametophytes (*E. kurome*)-treated group was significantly lower than that of DW-, sporophytes (*E. kurome*), or filamentous alga-treated groups (Fig. 1B). The level of serum triacylglycerol of the gametophytes (*E. kurome*)-treated group was lower than that of the DW-treated group (Fig. 1B). In contrast, there was no significant difference in the serum level of cholesterol between groups (Fig. 1B).

It is reported that leptin can act as a negative regulator of regulatory T cells (Treg) (14). By analyzing 50,000 lymphocytes pooled from each group, we found an interesting feature of lymphocyte subpopulation in the above experiment using *db/db* mice, namely, that percentage of Treg cells (CD4^+^CD25^+^ T cells) in lymphocytes of the group administered with gametophytes of *E. kurome* was lower than that of the group administered with DW (Table 3, Exp. 1).

**Figure 2:**
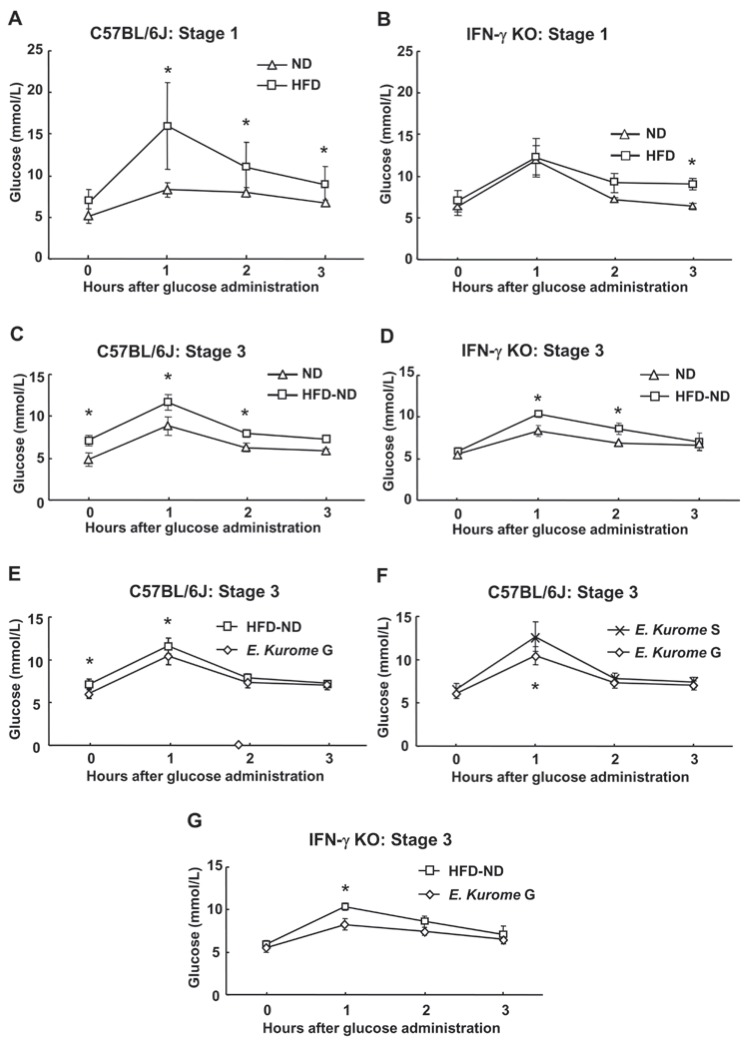
Effects of algae on the oral glucose tolerance test in prediabetic C57BL/6J or IFN-γ KO mice induced by a high fat diet. Blood glucose concentrations during oral glucose tolerance tests (2 g/kg body weight) following 18 hours of fasting in either C57BL/6J or IFN-γ KO mice fed normal diet (ND: Δ) or high fat diet (HFD: □). At stage 3, administration of HFD for 12 weeks and ND for 3 weeks is expressed as HFD-ND: □. Experiments were done at the end of each indicated stage shown in Table 1. A. Stage 1, C57BL/6J. B. Stage 1, IFN-γ KO. C. Stage 3, C57BL/6J, D. Stage3, IFN-γ KO. E-G. Effect of gametophytes of *E. kurome* on OGTT in either C57BL/6J (B6) or IFN-γ KO mice. Either gametophytes (*E. kurome* G: ◊) or sporophytes (*E. kurome* S: X) of *E. kurome* were administered to these HFD-ND-fed mice. E. Stage 3: HFD-ND vs. *E. kurome* G, C57BL/6J. F. Stage 3: *E. kurome* S vs. *E. kurome* G. C57BL/6J. G. Stage 3: HFD-ND vs. *E. kurome* G, IFN-γ KO. Results are means ± S.D. (C57BL/6J: n=8, IFN-γ KO: n=5). An ANOVA was performed and significance was determined using Tukey-Kramer’s post hoc test. Asterisks indicate that there are significant differences at each point between groups (*P*<0.05).

Then, we asked if gametophytes of *E. kurome* were effective in down-regulating blood glucose in prediabetic wild type mice whose condition was caused by a high fat diet. High fat diet (HFD) was administered to C57BL/6J and C57BL/6J-IFN-γ KO mice, C57BL/6J-*Ifng^tm1Ts^* for 8 weeks (Table 1). We used IFN-γ KO mice, because it is reported that leptin stimulates the proliferation of IFN-γ producing cells (13, 14). At the end of stage 1, we found a higher level of blood glucose one hour after glucose administration in the oral glucose tolerance test (OGTT) of IFN-γ KO mice compared with that of parental C57BL/6J mice (ND: Fig. 2A, B). Through using OGTT, we confirmed that HFD caused not only higher body weight but also prediabetic status in both strains of mice (Table 2, Fig. 2A, B). The effect of HFD was much greater in C57BL/6J mice. We found that one hour after administering glucose, the level of blood glucose was more than two times higher in C57BL/6J mice as compared with the ND-administered group (Fig. 2A). In contrast, the effects of HFD were not seen one hour after glucose administration in IFN-γ KO mice at the end of stage 1 (Fig. 2B). A higher level of glucose was observed three hours after glucose administration in HFD-treated group (8.96 ± 0.7 mmol/L) compared with ND-treated group (6.32 ± 0.41 mmol/L) in IFN-γ KO mice (Fig. 2B).

**Table 2:** Effects of algae on body weights of C57BL/6J and IFN-γ KO mice

Weeks	0	4	8	12	15	18

Stages	Start	Stage 1	End of Stage 1	End of Stage 2	End of Stage 3	End of Stage 4
Group 1	17.5 ± 0.6	19.1 ± 0.9^a^	20.9 ± 1.3^a^	22.9 ± 0.9^a^	23.8 ± 0.9^a^	23.8 ± 1.2^a^
Group 2	18.3 ± 0.6	21.9 ± 1.1	27.7 ± 1.5	34.8 ± 3.1	26.8 ± 1.6	36.6 ± 2.0
Group 3	18.0 ± 0.6	21.7 ± 1.5	25.5 ± 2.6	32.3 ± 5.0	26.5 ± 2.3	34.8 ± 3.9
Group 4	19.1 ± 0.9	24.2 ± 2.2	29.5 ± 3.5	35.4 ± 3.6	27.5 ± 2.3	35.9 ± 4.0
Group 5	18.4 ± 0.4	21.9 ± 0.8	25.9 ± 1.8	30.5 ± 3.6	26.5 ± 1.7	34 ± 2.7
Group 6	18.5 ± 0.9	22.0 ± 2.5	26.3 ± 4.1	31.9 ± 5.4	26.5 ± 1.8	32.6 ± 5.1
Group 7	19.3 ± 0.7	21 ± 0.8	21.9 ± 0.4	23.9 ± 0.7^a^	24.0 ± 0.6	24.2 ± 0.7^a^
Group 8	22.5 ± 3	24.9 ± 5.3	30.0 ± 7.4	35.2 ± 9.4	28.0 ± 5.4	34.6 ± 8.9
Group 9	23.5 ± 2.6	24.3 ± 3.0	28.0 ± 5.1	32.3 ± 8.1	26.6 ± 4.1	32.4 ± 7.3

Average body weight (gram, mean ± S.D.) of each group shown in Table 1.

a A significant difference between the ND group and HFD group (Group1 *vs.* Group 2, Group7 *vs.* Group 8: *P*<0.05, one-way ANOVA).

**Figure 3:**
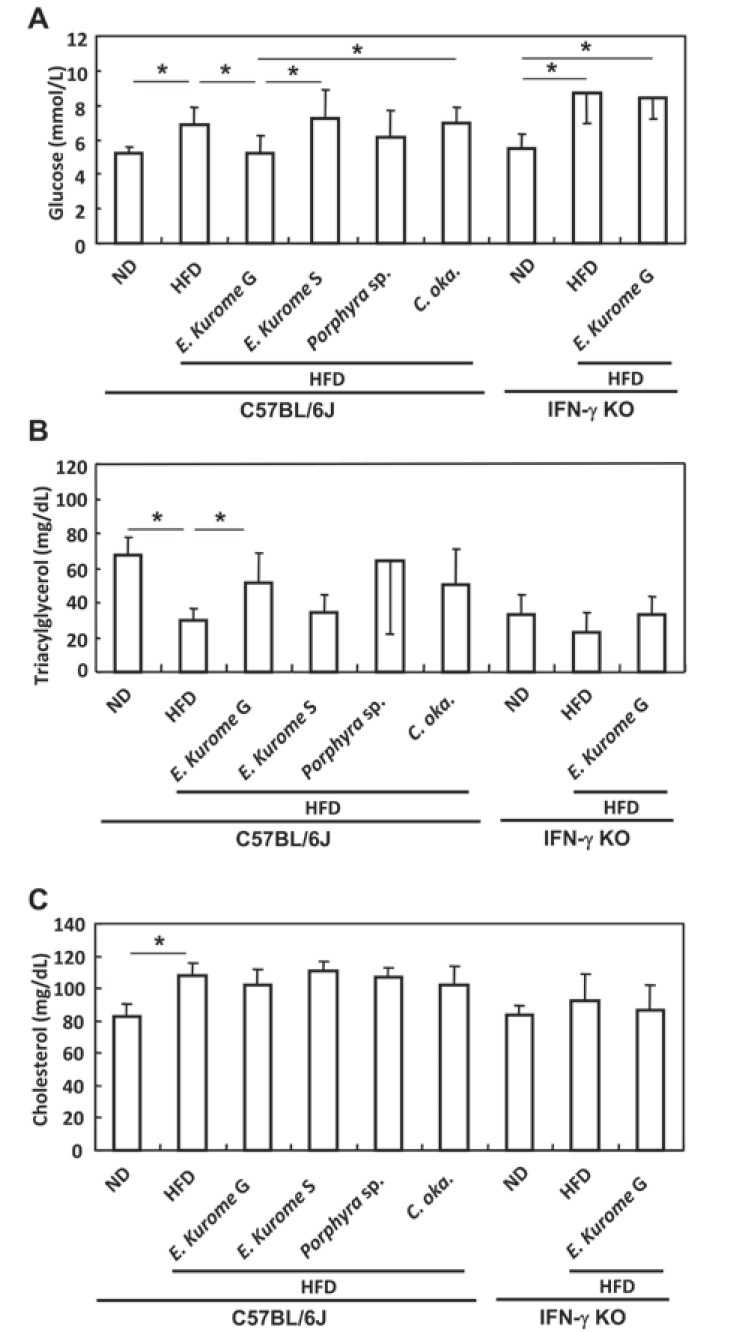
Effects of algae on the serum levels of glucose, triacylglycerol, and cholesterol in prediabetic C57BL/6J or IFN-γ KO mice induced by a high fat diet. Gametophytes of *E. kurome*, sporophytes of *E. kurome*, red alga *Porphyra* sp., and brown alga *C. oka*. (*Cladosiphon okamuranus*) were administered to C57BL6/J or IFN-γ KO mice orally. Diets and algae homogenates were administered as summarized in Table 1. Serums were prepared at the stage 4. A. Serum levels of glucose. Gametophytes but not sporophytes of *E. kurome* were effective in down-regulating the serum glucose level compared with the HFD group; B. Serum levels of triacylglycerol. Gametophytes but not sporophytes of *E. kurome* were effective in up-regulating the serum triacylglycerol level compared with the HFD group; C. Serum levels of cholesterol. HFD significantly up-regulated the serum level of cholesterol. Results are expressed as average ± S.D. The mouse No. of each group is also described in Table 1. Asterisks indicate that there are significant differences between groups paired by a line. *One way ANOVA, *P*<0.05.

Administration of gametophytes of *E. kurome* for four weeks to HFD-fed mice did not have any effects on the OGTT in either C57BL/6J or C57BL/6J-IFN-γ KO mice at stage 2 (data not shown). After confirming that administration of gametophytes of *E. kurome* together with HFD do not have any effect on OGTT, we tried to evaluate the effect after administering them together with ND. Three weeks after switching the food from HFD to ND, both strains of mice were still prediabetic (Table 1, Fig. 2C, D). Blood glucose two hours after glucose administration to C57BL/6J and C57BL/6J-IFN-γ KO were 7.91 ± 0.46 mmol/L and 8.59 ± 0.68 mmol/L, respectively. At stage 3, gametophytes but not sporophytes of *E. kurome* were effective in the OGTT compared with the control group in C57BL/6J mice (Table 1, Fig. 2E, F). The value of blood glucose 2 hours after glucose administration in the group treated with gametophytes of *E. kuome* was 7.32 ± 0.63 mmol/L and that of HFD-ND was 7.91 ± 0.46 mmol/L. Although there was no significant difference at this point between these two groups, fasting plasma glucose was significantly reduced (HFD-ND: 7.12 ± 0.62 mmol/L, *E. kurome* G: 6.05 ± 0.54 mmol/L). As a result, gametophytes of *E. kurome* improved the glucose tolerance partially. *Porphyra* species and *C. okamuranus* did not have any effects on OGTT in C57BL/6J mice (data not shown). Gametophytes of *E. kurome* were also effective in the OGTT compared with the control (HFD-ND) group in IFN-γ KO mice (Fig. 2G). To maintain the prediabetic status, we changed the food from ND to HFD again and continued to administer homogenized algae to mice. Ten days after changing the food in stage 4, we measured the serum glucose, triacylglycerol, and cholesterol after fasting the mice for 18 hours. Among the algae homogenate examined, only gametophytes of *E. kurome* were effective in down-regulating the blood glucose (Fig. 3A). In contrast, gametophytes *E. kurome* did not have any effect on the serum level of glucose in IFN-γ KO mice (Fig. 3A). Gametophytes of *E. kurome* did not down-regulate the serum level of triacylglycerol in either strain of mice (Fig. 3B). Although it was significantly higher than those of the groups of DW (HFD), it was a little less than the ND group on average in C57BL/6J mice (Fig. 3B). Although HFD up-regulated the serum level of cholesterol, no algae homogenate had a significant effect on it in either strain of mice (Fig. 3C).

**Figure 4:**
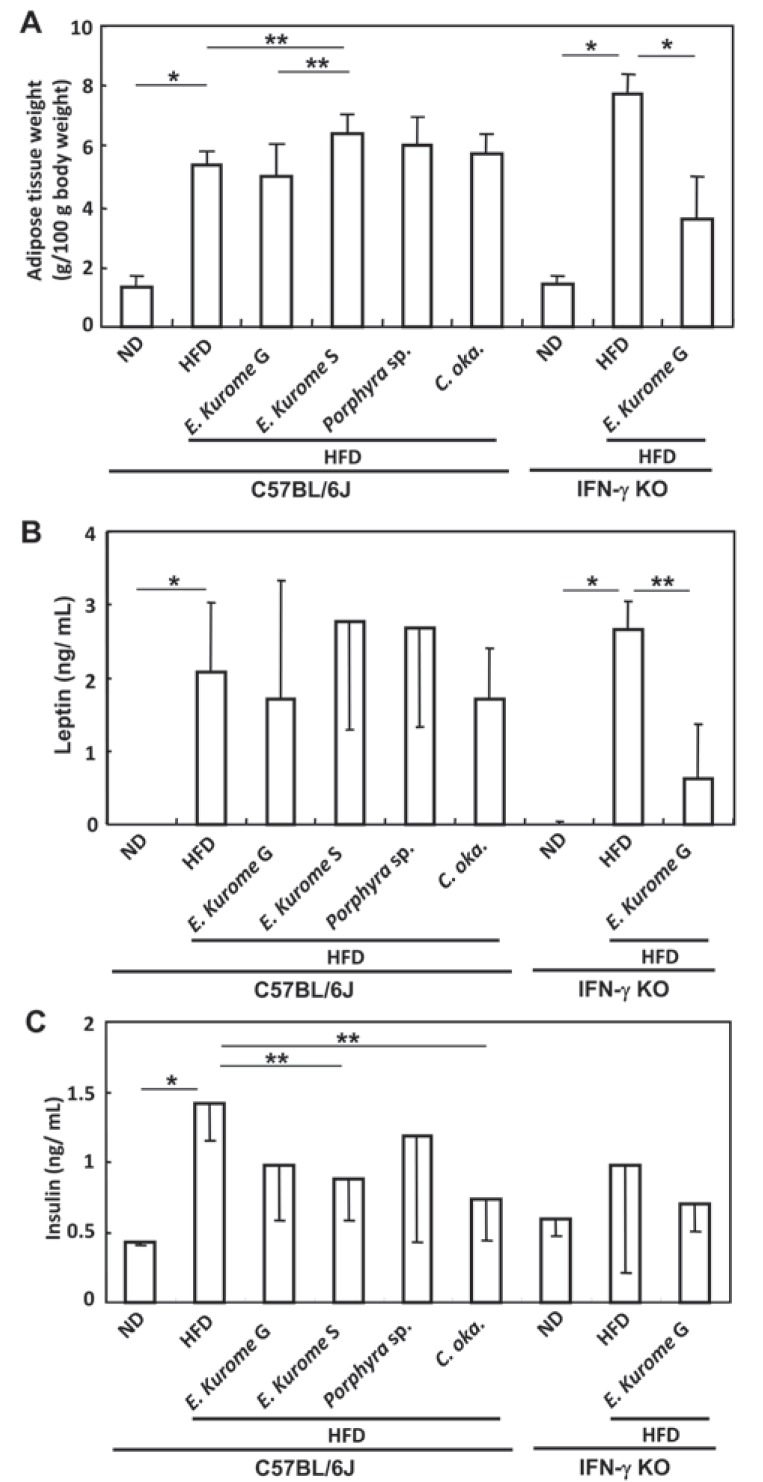
Effects of edible algae on the development of adipose fat tissue and the serum levels of leptin and insulin. Edible algae were administered to prediabetic C57BL/6J and IFN-γ KO mice as described in Table 1. A. At the end of the stage 4, adipose tissue around the uterus was measured. Results are expressed as mean adipose tissue weight (g/100 g body weight) ± S.D; B. Serum levels of leptin were measured. In the ND groups of either C57BL/6J or IFN-γ KO mice, serum levels of leptin were less than 50 pg/mL, that is the detection limit of this ELISA; C. Serum levels of insulin were measured. Results (B and C) were expressed as mean (ng/mL) ± S.D. Asterisks indicate that there are significant differences between groups paired by a line. *Tukey-Kramer’s post hoc test, *P*<0.05. **One way ANOVA, *P*<0.05.

We measured the weight of adipose fat tissue around the uterus at the end of stage 4. Adipose fat tissue accumulated in the groups of HFD-fed mice in both C57BL/6J and IFN-γ KO mice (Fig. 4A). Sporophytes but not gametophytes of *E. kurome* augmented adipose fat tissue slightly compared with the HFD group in C57BL/6J mice. The adipose tissue weight of the group administered with gametophytes was significantly reduced compared with that administered with HFD alone in IFN-γ KO mice. There was no significant difference in body weight among HFD-fed groups in either C57BL/6J or IFN-γ KO mice, although mice in HFD groups had higher body weights compared with those in ND groups in both strains of mice as described in Table 2.

The serum level of leptin of each group closely corresponded to the level of adipose fat tissue (Fig. 4A, B). Insulin resistance was observed in prediabetic HFD-fed mice in overnight-fasted C57BL/6J but not IFN-γ KO mice (Fig. 4C). Gametophytes of *E. kurome*, sporophytes of *E. kurome* and *C. okamuranus* had a tendency to ameliorate the insulin resistance in C57BL/6J mice (Fig. 4C).

As described above, Treg cells were decreased by the treatment of gametophytes of *E. kurome* in *db/db* mice. We measured the amounts of Treg cells in C57BL/6J mice and IFN-γ KO mice after the treatment with gametophytes of *E. kurome* at stage 4. We observed a slight decrease of CD4^+^CD25^+^ cells in lymphocytes from both gametophytes or sporophytes of *E. kurome*-treated group in C57BL/6J mice compared with DW- or *Prophyra* sp.- treated group (Exp. 2). In IFN-γ KO mice, the percentage of CD4^+^CD25^+^ cells in ND group is higher than those of C57BL/6J and *db/db* mice (Table 3). Interestingly, we found lower percentage of CD4^+^CD25^+^ cells in lymphocytes from gametophytes of *E. kurome*-treated group than that of DW-treated group in IFN-γ KO mice (Table 3). This significant decrease of CD4^+^CD25^+^ cells in the group treated with gametophytes of *E. kurome* was also confirmed by analyzing CD4^+^FOXP3^+^ cells and CD25^+^FOXP3^+^ cells (FOXP3: forkhead-winged-helix transcription factor, data not shown).

**Table 3:** Effects of algae on the Treg cells

**Exp. 1.** Percentages of CD4^+^CD25^+^ T cells among total lymphocytes in *db/db* mice						

	**No treat**	**DW**	***E. kurome* G**	***Ulva* sp.2**	**Fil. Alga**	**S. *pacifica***
	
CD4^+^CD25^+^ T cells (%)	3.01	2.73	1.94	2.41	2.52	3.21

**Exp. 2.** Percentages of CD4^+^CD25^+^ T cells among total lymphocytes in C57BL/6J mice						

	**Group 1 ND (CE-2)**	**Group 2 HFD + DW**	**Group 3 HFD + gametophytes *E. kurome***	**Group 4 HFD + sporophytes *E. kurome***	**Group 6 HFD + *Porphyra* sp**	
	
CD4^+^CD25^+^ T cells (%)	2.93	2.76	2.50	2.54	3.14	

**Exp. 3.** Percentages of CD4^+^CD25^+^ T cells among total lymphocytes in IFN-γ KO mice						

	**Group 7 ND (CE-2)**		**Group 8 HFD + DW**		**Group 9 HFD + gametophytes *E. kurome***	
	
CD4^+^CD25^+^T cells (%)	4.02		3.09		2.13	

No treat, without any oral administration. In Exp. 1 described in Fig. 1A, two weeks before the sampling of sera, lymphocytes were collected from peripheral blood of each mouse. Lymphocytes pooled from each group were stained with PerCP-anti-CD4 Ab and FITC-anti-CD25 Ab, and fifty thousands cells were analyzed as described in Materials and methods. Fil. Alga: filamentous blue-green alga; Exp. 2 and Exp. 3: At the Stage 4 of Table 1, one day before being sacrificed, lymphocytes were collected from peripheral blood of each mouse. Lymphocytes pooled from each group were stained with PerCP-anti-CD4 antibodies and PE-anti-CD25 Ab, and fifty thousands cells were analyzed as described in Materials and methods.

## DISCUSSION

In some parts of Japan, it has been believed that *Ecklonia* species could improve the property of blood. We have studied algae that have the potential to down-regulate the blood glucose by taking them as food. At first, we examined the edible algae collected around Shikoku Island and Okinawa Island, Japan, and *S. pacifica* cultured in Kona, Hawaii Island. We administered these algae homogenate to leptin receptor deficient *db/db* mice that are known as model mice of diabetes type 2 and evaluated them in terms of the down-regulating ability of blood glucose. We also administered these algae to HFD-fed C57BL/6J mice that were reportedly a robust and efficient model for impaired glucose tolerance test and that of early type 2 diabetes which may be used for studies on pathophysiology and the development of new treatments (20, 21). Although the wild type mouse strain varies in their ability to secrete insulin in response to HFD (22), 8 weeks-HFD-fed C57BL/6J mice are still quite a good model of type 2 diabetes (23). Since it is reported that 2 g/kg glucose administered orally following 6 hours of fasting is the best way to assess glucose tolerance in mice (23), we administered 2 g/kg glucose orally following the fasting according to the manufacturer’s protocol. In terms of fasting hours, however, we chose 18 hours fasting, because it is reported that overnight fasting nearly depletes glycogen stores in the mouse and is useful for studies where the focus is on glucose utilization (24). Indeed, we focused on the role of IFN-γ on glucose utilization. As discussed below, IFN-γ is certainly involved in the regulation of glucose usage.

Interestingly, we found that gametophytes but not sporophytes of *E. kurome* were effective in down-regulating blood glucose in both *db/db* mice and prediabetic C57BL/6J mice, suggesting that active compounds are enriched in the gametophytes. Since we used a mixture of female and male gametophytes, active compounds may exist in either gametophyte. These compounds are not likely to reduce the absorption of foods, because body weights of mice did not change significantly among HFD-fed groups, though different algae were administered (Table 2).

One of the well-studied active compounds extracted from edible brown algae to down-regulate blood glucose is fucoxanthin. It down-regulated not only the level of blood glucose and plasma insulin but also the expression level of leptin and TNF-α mRNAs in white adipose tissue of the diabetes/obesity mouse KK-*A^y^* (25). It has also been reported that supplementation of fucoxanthin-rich seaweed extract reduced white adipose tissue, plasma and hepatic triacylglycerol, and/or cholesterol compared to the HFD-fed C57BL/6J mice (26). Furthermore, Woo *et al*. reported that fucoxanthin supplementation reduced the hepatic lipogenic enzymes, glucose-6-phosphate dehydrogenase, malic enzyme, fatty acid synthase, and phosphatidate phosphohydrolase, and enhanced the activity of ß-oxidation (27). They also reported that fucoxanthin suppressed the following cholesterol regulating enzymes: 3-hydroxy-3-methylglutaryl coenzyme A reductase and acyl coenzyme A: cholesterol acyltransferase.

Although fucoxanthin is extracted from sporophytes of various brown algae such as *Undaria pinnatifida, Sargassum fulvellum, Laminaria japonica, Hiziki fusiformis,* there is no study which has compared the quantity of fucoxanthin in the life cycle stage of brown algae. Since fucoxanthin is rich in brown algae and *C. okamuranus* did not decrease the blood glucose, it is unlikely that fucoxanthin is one of the major compound in gametophytes of *E. kurome* which down-regulates the blood glucose.

Phlorotannins such as fucosterol (28) in brown algae are reported to be anti-diabetic. Fraction enriched in phlorotannins is also reported to have the capacity to inhibit α-amylase and α-glucosidase activities, suggesting the anti-diabetic properties of these components (29). Furthermore, Xu *et al*. reported that phlorotannins of *E. kurome* improve the tolerance and decreased the fasting blood glucose and insulin levels, fructosamine and glycoalbumin levels compared with the control group (30). Nishino *et al*. reported that *E. kurome* has a highly branched, new type of fucan sulfate containing a backbone of (1-3)-linked L-fucosyl residues having sulfate groups mainly attached to C-4 and it has a potent anticoagulant activity (31).

The question of whether or not there are active compounds selectively produced or accumulated in gametophytes of *E. kurome* remains to be asked. We think it is possible that gametophytes accumulate triacylglycerol or carbohydrates by converting glucose as energy sources before fertilization, because it is common for plants to accumulate energy sources at a certain stage of the life cycle such as when producing seeds. This hypothesis may be supported by the fact that gametophytes but not sporophytes of *E. kurome* recovered the reduced level of triacylglycerol by HFD to the level of ND group in C57BL/6J mice. This effect of gametophytes of *E. kurome* was not observed in IFN-γ KO mice (Fig. 3B). In *db/db* mice, the serum level of triacylglycerol was decreased by administrating gametophytes but not sporophytes of *E. kurome*. These results suggest that both leptin and IFN-γ are involved in the regulation of the metabolism of glucose and triacylglycerol.

It is reported that T cells from obese adipose tissue produce more IFN-γ than those from controls (32). Recently, Wong *et al.* reported that IFN-γ KO mice had improved glucose tolerance with a reduced rate of glucose appearance and increased insulin sensitivity due to greater suppression of endogenous glucose output, which was associated with decreased hepatic glucose-6 phosphatase activity after administrating a low-fat chow diet over 20 weeks (33). They concluded that global deletion of *IFN-γ* in mice resulted in reduced body weight associated with negative energy balance, improved glucose tolerance, and hepatic insulin sensitivity. We have also observed the improved glucose tolerance in HFD-fed IFN-γ KO mice compared with HFD-fed C57BL/6J mice (Fig. 2A, B).

It is suggested that leptin may be the key link between nutritional status and an optimal immune response (13). De Rosa *et al.* reported that leptin down-regulates the Treg cells and stimulates the proliferation of IFN-g secreting Th1 cells (14). In *db/db* mice, Treg cells were reduced in the group administered with gametophytes of *E. kurome* (Table 3). This may suggest that gametophytes of *E. kurome* may replace the function of leptin, in part, in regulation of Treg cells. However, serum level of IFN-γ was not increased in the group administered with gametophytes of *E. kurome* (data not shown).

In order to clarify the role of Treg cells in the down-regulation of blood glucose, we examined the effects of *E. kurome* on the population of Treg cells in C57BL/6J and IFN-γ KO mice. Although there was a slight down-regulation of Treg cells in the gametophytes *E. kurome*-administered group compared with the control HFD- or *Porphyra* sp.-administered group, there was no significant difference between the gametophytes-administered group and the sporophytes-administered group in C57BL/6J mice (Table 3). Although gametophytes of *E. kurome* decreased Treg cells, they had no effect on the serum level of glucose at the stage 4 in IFN-γ KO mice (Fig, 3A), suggesting that Treg cells are not directly involved in the regulation of glucose in these mice. However, Treg cells may be engaged in the regulation of adipose fat tissue and leptin production (Table 3 and Fig. 4A, B).

Leptin is not a simple satiety signal to prevent obesity in times of energy excess. Falling leptin concentration is a critical signal that initiates the neuroendocrine response to starvation, including limiting procreation, decreasing thyroid thermogenesis, and increasing secretion of stress steroids, which together are likely to have survival value during prolonged nutritional deprivation (34). On the other hand, proinflammatory cytokine IFN-γ produces fever and enhances thermogenesis suggesting the intimate relation between leptin and IFN-γ.

Another important point is that germ-free C57BL/6J mice are resistant to HFD-induced insulin resistance and have altered cholesterol. Rabot *et al*. have demonstrated that lower calorie consumption and increased lipid excretion contributed to the obesity-resistant phenotype of germ-free and HFD-fed mice (35). They also revealed that insulin sensitivity and cholesterol metabolism are metabolic targets influenced by the gut microbiota. Mazmanian *et al*. reported that colonization with wild-type *Bacteroides fragilis* alone is sufficient to correct the defect in IFN-γ expression in germ-free mice, with levels nearly as high as those in conventional mice (36). This effect of IFN-γ expression depends on the bacterial polysaccharides of *Bacteroides fragilis*.

These results may suggest that gametophytes of *E. kurome* are effective in regulating the metabolism through the manipulation of balance among cytokines including IFN-γ or leptin, resulting in the down-regulation of blood glucose.

In conclusion, gametophytes of *E. kurome* are useful in down-regulating blood glucose and are good candidates to be used as an alternative medicine, because they are one stage of an edible alga. Although anti-diabetic effects of gametophytes of edible algae other than *E. kurome* still remain to be studied, gametophytes may be enriched in the regulatory compounds engaged in regulation of metabolism. Furthermore, these bioactive molecules may function to reduce the blood glucose and white adipose tissue by regulating immune system and endocrine system associated with life cycle and cell differentiation. We also found that IFN-γ is engaged in the regulation of blood glucose.
